# Extracellular Vesicles from Human Papilloma Virus-Infected Cervical Cancer Cells Enhance HIV-1 Replication in Differentiated U1 Cell Line

**DOI:** 10.3390/v12020239

**Published:** 2020-02-21

**Authors:** Sabina Ranjit, Sunitha Kodidela, Namita Sinha, Subhash Chauhan, Santosh Kumar

**Affiliations:** Department of Pharmaceutical Sciences, University of Tennessee Health Science Center, Memphis, TN 38163, USA; Sabina.Ranjit@STJUDE.ORG (S.R.); skodidel@uthsc.edu (S.K.); nsinha2@uthsc.edu (N.S.); subhash.chauhan@utrgv.edu (S.C.)

**Keywords:** HIV, HPV, extracellular vesicles, CYPs, oxidative stress, cervical cancer

## Abstract

In the current study, we hypothesized that extracellular vesicles (EVs) secreted from human papilloma virus (HPV)-infected cervical cancer cells exacerbate human immunodeficiency virus (HIV)-1 replication in differentiated U1 cell line through an oxidative stress pathway. To test the hypothesis, we treated an HIV-1-infected macrophage cell line (U1) with HPV-infected Caski cell culture supernatant (CCS). We observed a significant increase in HIV-1 replication, which was associated with an increase in the expression of cytochrome P450 (CYPs 1A1 and 2A6) in the CCS-treated U1 cells. Furthermore, we isolated EVs from CCS (CCS-EVs), which showed the presence of CYPs (1A1, 2A6), superoxide dismutase 1 (SOD1), and HPV oncoproteins HPV16 E6. CCS-EVs when exposed to the U1 cells also significantly increased HIV-1 replication. Treatment of antioxidant, CYP1A1 and CYP2A6 inhibitors, and chemodietary agents with antioxidant properties significantly reduced the CCS and CCS-EVs mediated HIV-1 replication in U1 cells. Altogether, we demonstrate that cervical cancer cells exacerbate HIV-1 replication in differentiated U1 cell line via transferring CYPs and HPV oncoproteins through EVs. We also show that the viral replication occurs via CYP and oxidative stress pathways, and the viral replication is also reduced by chemodietary agents. This study provides important information regarding biological interactions between HPV and HIV-1 via EVs leading to enhanced HIV-1 replication.

## 1. Introduction

Human papillomavirus (HPV) and human immunodeficiency virus (HIV) are both infections that can be transmitted sexually. Different types of HPV strains exist but only two types,16 and 18, cause most of the HPV-related cancers. There are about 44,000 HPV-associated cancers diagnosed in the United States each year [1]. Among HPV associated cancers, cervical cancer is the most common among women. The average number of cervical cancers caused by HPV per year is reported to be 12,015 [1]. Interestingly, HIV-1-infected women have a high prevalence of human papilloma virus (HPV) infection, particularly types 16 and 18 that cause cervical cancer [2,3]. Epidemiological data suggest that the incidence of cervical cancer in HIV-1-infected women is approximately 5- to 10-times higher compared to uninfected women [4]. Cervical cancer is also categorized into acquired immunodeficiency syndrome (AIDS)-defining illness [5]. HIV-1 increases HPV infectivity by disrupting the tight junctions surrounding the epithelial cells. HIV-1 further enhances the ability of HPV to develop precancerous lesions and ultimately cervical cancer by promoting immunosuppression and upregulation of HPV oncogene [4]. However, whether HPV infection has a reciprocal effect on HIV-1 pathogenesis is largely unknown. Therefore, there is a need to examine the biological interactions between HPV and HIV-1 to find better preventive and treatment strategies to reduce HIV-1 pathogenesis in HIV-1/HPV coinfected individuals.

Evidences from previous reports show that cervical cancer cells constantly undergo oxidative stress [6,7,8]. Clinical samples from cervical cancer patients reveal a higher reactive oxygen species (ROS) level, higher oxidative damage, and a lower level of antioxidants, compared to samples from healthy individuals [9,10,11]. In vitro experiments also suggest a high level of ROS, profound downregulation in the genes associated with antioxidant proteins, such as superoxide dismutase 1 (SOD1), SOD2, SOD3, peroxiredoxin 1 (PRDX1), PRDX2, glutathione S-synthetase (GSS), and glutathione peroxidase 6 (GPX6), and high presence of Poly [ADP-ribose] polymerase 1 (PARP1), a marker of oxidative stress-induced DNA damage in cervical carcinoma-derived Caski cells [7]. HPV oncoproteins E6 and E7 stimulate the degradation of tumor-suppressor p53 protein [12], causing uncontrolled cell growth. The p53 protein, which primarily attributes to apoptosis and genomic stability, is also reported to have an antioxidant role [13]. Downregulation of p53 suppresses its antioxidant potential and renders the cells vulnerable to oxidative damage. Furthermore, the expression of HPV E6 and E7 oncoproteins alone is sufficient to cause oxidative stress. Marullo et al. demonstrated that HPV E6 and E7 proteins generate a chronic oxidative response in host cells via NADPH oxidase 2 (NOX2) activation [14]. E6 expression also downregulates SOD2 and glutathione peroxidase [14]. The suppression of antioxidant activity of these antioxidants could be another mechanism by which HPV generates ROS in the host cells.

Similar to other cells, HPV-infected cervical cells also secrete small nanosized extracellular vesicles (EVs) [15,16]. EVs originate from invagination of the lumen of early endosomes. As the endosomes reach the later phase of their development, these invaginations bud off forming hundreds of intraluminal vesicles. These vesicles are released into the extracellular matrix of the cells through fusion into the cell membrane [17]. EVs carry a wide variety of cellular proteins, lipids, cytokines, mRNA, miRNA, and DNA from their originating cells to other cells via blood circulation. The role of EVs in intra- and intercellular communications and transport is well supported by many studies [18]. In HPV-infected cells, the virus hijacks the host EVs system, thereby modulating both the contents and number of EVs released from HPV-infected cervical cells [16,19]. EVs from HPV-infected cervical cells are known to transport various cellular factors to recipient cells [19,20]. Therefore, it is possible that HPV-infected cells release oxidative stress factors into EVs and transfer them to other cells via the blood stream.

Oxidative stress-induced viral replication has also been observed in HIV-1 infection [21,22]. We recently demonstrated that oxidative stress generated via cytochrome P450 (CYP)-mediated metabolism of tobacco constituents, especially cigarette smoke condensate (CSC) and benzo(a)pyrene (BaP), triggers HIV-1 replication in monocytes and macrophages [23,24]. We showed that ROS thus generated, activate the nuclear transcription factor NF-κB, thereby inducing transcription of HIV-1. Further, we showed the role of EVs carrying CYP and antioxidant enzymes (AOEs) in cytotoxicity and HIV-1 replication [24]. In the current study, we study the role of EVs as mediators for intercellular communication between HPV-infected cervical cancer cells and differentiated U1 cell line latently infected with HIV-1. Our findings demonstrate that cervical cancer cells release EVs containing oxidative stress factors into differentiated U1 cell line and induce HIV-1 replication.

## 2. Materials and Methods

### 2.1. Cell Culture and Treatment

Caski cells contain about 600 copies per cell of integrated HPV16 sequences, which cause cancer-related biological effects, such as deregulation of P53. Due to this, Caski cell line is considered a model cell line and widely used in cervical cancer studies. Moreover, American Type Culture Collection (ATCC) confirmed that this cell line is positive for the presence of human papilloma virus (HPV) viral sequences via PCR.

We cultured Caski cells in Roswell Park Memorial Institute (RPMI) 1640 media containing 10% fetal bovine serum (FBS) and penicillin. We seeded about 2.5 million cells in a T-75 flask, cultured them for 3 days, and collected the culture supernatant on the fourth day. For EVs isolation, we cultured the Caski cells in an RPMI media containing 10% EVs-free FBS and penicillin.

U1 cells, which are U937 cells chronically infected with HIV-1, were obtained from the NIH AIDS Reagent Program (Germantown, MD, USA). The cells were cultured in Roswell Park Memorial Institute (RPMI) 1640 media containing 10% FBS and penicillin. To differentiate the U1 cells into macrophages, 0.8 million cells were seeded in 1.5 mL of media containing 100 nM phorbol 12-myristate 13-acetate (PMA) in each well of a 6-well plate. After 3 days, the media containing PMA and non-adherent cells was removed and the differentiated cells were washed with phosphate buffer saline (PBS). The cells were treated with 250 µL of Caski cell culture supernatant (CCS) every 24 h for 4 days and EVs derived from 1 mL of CCS (~42 µg of EVs protein) for 4 days.

### 2.2. EVs Isolation and Characterization

We isolated EVs from Caski cell culture media using Invitrogen-Total EVs Isolation (from cell culture media) kit (Life Technologies, Grand Island, NY, USA). Briefly, samples were run through a 0.22 µm filter to remove any particles, which are >0.22 µm and the filtered supernatant was centrifuged at 2000× *g* for 30 min to remove any cellular debris present. Total EVs isolation reagent was added to the cell culture supernatant in a 1:2 ratio and the mixture was incubated overnight at 2–8 °C. The next day, the mixture was centrifuged at 10,000× *g*, 2–8 °C for 1 h to obtain the EV pellets. We understand that ultracentrifugation is the gold standard method to isolate purified EVs, but it requires a large volume of starting material and has low extraction efficiency [25,26]. Therefore, we used Invitrogen-Total EVs Isolation kit, which requires less media, but at the same time has high extraction efficiency [25,26]. In our previous report, the EVs isolated from the media of HIV-1-infected monocytes/macrophage cells using ultracentrifugation and Invitrogen-Total EVs Isolation commercial kit did not show significant differences in terms of physical and biochemical characteristics [24]. The commercial kit also showed minimal and insignificant contamination from the HIV particles.

We followed the international Society for Extracellular Vesicles (ISEV) guidelines for extracellular vesicles characterization. To confirm the identity of EVs, we performed Western blotting using antibodies against specific EVs marker proteins such as CD63, CD81, Alix, and CD9, as well as acetylcholine esterase activity. We also used actin and glyceraldehyde 3-phosphate dehydrogenase (GAPDH), which are the major housekeeping proteins in cells and are generally absent in EVs. The EVs were further characterized by measuring their size using Zetasizer Nano-ZS (Malvern Instruments Inc, Malvern, UK) as described previously [24]. We also quantified and further validated the EVs by measuring acetylcholinesterase activity using the fluorescent Amplex^®^ Red Acetylcholine/Acetylcholinesterase Assay Kit (Molecular Probes, Invitrogen, Grand Island, NY, USA) as described previously [27]. Briefly, EVs isolated from 600 μL media was resuspended in 100 μL of 1X reaction buffer and incubated with a working solution of 400 μM Amplex Red reagent containing 2 U/mL horseradish peroxidase (HRP), 0.2 U/mL choline oxidase, and 100 μM acetylcholine. A 0.2 U/mL acetylcholinesterase was used as a positive control. The fluorescence intensity was measured in a microplate reader using excitation of 545 nm and emission at 590 nm.

### 2.3. Viral Load

We collected the supernatant from U1 cells treated with Caski cell supernatant or Caski cell-derived EVs. HIV-1 viral p24 antigen level was measured in the U1 cell culture supernatant to access the viral load, using the HIV-1 p24 Antigen ELISA kit (Zeptometrix Corporation, Buffalo, NY, USA) according to the manufacturer’s protocol. Briefly, the kit is comprised of monoclonal antibody- coated microwells, which specifically bind the HIV-1 p24 antigen in the added samples. The captured antigen was incubated with biotin conjugated human anti-HIV-1 antibody at 37 °C for 1 h, followed by incubation with enzyme, streptavidin-peroxidase, and tetramethylbenzidine substrate at 37 °C and room temperature/dark, respectively, for 30 min each. The reaction of the enzyme with the substrate developed a blue color, the absorbance of which was measured at 450 nm to determine the p24 level. The optical density of the samples was compared against the standard curve.

### 2.4. Measurement of ROS

ROS was measured in U1 cells after CCS or CCS-EVs (EVs isolated from CCS) treatment. ROS was quantified by flow cytometry using the fluorescence dye 5-(and-6)-chloromethyl 2′,7′- dichlorodihydrofluorescein diacetate (CM-H2DCFDA) (Life Technologies, Oregon, USA). The treated cells were thoroughly washed with PBS and resuspended in 1 mL of PBS containing 2% FBS supplemented with 2–5 μL of CM-H2DCFDA. The cells were then incubated at room temperature in the dark for 30 min and subsequently washed and resuspended in 300 µL of PBS containing 2% FBS. Dichlorodihydrofluorescein (DCF) emission at 525 ± 20 nm, which is proportional to the ROS generated in the cells, was detected by flow cytometry (ACEA, Biosciences Inc., San Diego, CA, USA), and NovoExpress software was used to analyze the data. The background fluorescence signal due to unlabeled cells was reduced and only the live healthy patch of cells was gated to calculate the mean fluorescence intensity of the dye.

### 2.5. Total Antioxidant Capacity

We measured antioxidant capacity of the CCS or CCS-EVs treated U1 cells using the Total Antioxidant Capacity Assay (TCA) Kit (Bio Vision, Milpitas, CA, USA). The assay was performed according to the manufacturer’s protocol and as described previously [24].

### 2.6. Cytotoxicity

We used the Pierce^TM^ LDH Cytotoxicity Assay Kit (Thermo Scientific, Rockford, IL, USA) to measure the cytotoxicity in U1 cells after exposure of Caski cell EVs. We followed the manufacturer’s protocol to conduct the assay. We performed the assay on culture supernatant obtained after the treatment. Dead cells release lactic acid dehydrogenase (LDH) enzymes from the cytosol into the culture supernatant through their damaged plasma membrane. The measure of LDH in the culture supernatant is proportional to the cytotoxicity suffered by the cells. The LDH kit is comprised of LDH reaction mixture containing lactate, NAD^+^, diaphrose, and terazolium salt. A catalytic reaction between the LDH in the medium and the reaction mixture generates a red color product, formazan, whose absorbance at 490 nm is directly proportional to the amount of LDH.

### 2.7. Apoptotic DNA Damage

We observed the DNA damage of the CCS or CCS-EVs treated U1 cells under fluorescent microscope using the Apoptag^®^ Iso Dual Fluorescence Apoptosis Detection kit (Millipore Sigma, Burlington, MA, USA). We followed the manufacturer’s protocol to perform the assay.

### 2.8. RNA and Protein Isolation

RNA was isolated using RNeasy Mini kit (250) (QIAGEN, Germantown, MD, USA), following the manufacturer’s protocol. The extracted RNA was quantified using Nanodrop 2000c Spectrophotometer (Thermo Fisher Scientific, Wilmington, DE 19810 USA) at 260 nm. To isolate the protein from the treated cells, 100 µL of RIPA buffer was added to the cell pellet. The cell suspension was sonicated for 30 s with pulse set at 4 and centrifuged at 13,000 rpm for 5 min. The supernatant containing the protein was collected, and protein quantification was done by using the BCA Protein Assay Kit (Thermo Fisher Scientific, Rockford, IL, USA).

### 2.9. Quantitative Reverse Transcriptase Polymerase Chain Reaction (RT-PCR)

We used RTPCR to calculate the relative mRNA fold expression level of CYPs and AOEs in U1 cells after treatment of Caski supernatant/Caski-derived EVs. Purified RNA (120 ng) was reverse transcribed to cDNA using a SimpliAmp Thermal Cycler (Applied Biosystems, Foster City, CA, USA). The cDNA was amplified in a Step-One Plus Real-Time PCR System (Applied Biosystems, Foster City, CA, USA) using the TaqMan Gene Expression kit (Applied Biosystems, Foster City, CA, USA). The 2-∆∆Ct method was used to calculate the relative mRNA fold expression of the genes, using glyceraldehyde 3-phosphate dehydrogenase (GAPDH) as an endogenous control. We used the following specific TaqMan TM probes (CYP1A1 (Hs01054794_m1), CYP2A6 (Hs00430021_m1), SOD1 (Hs00533490_m1), SOD2 (Hs00167309_m1), catalase (Hs00156308_m1), and PRDX6 (Hs00705355_s1)) and GAPDH as an endogenous control.

### 2.10. Western Blotting

We used Western blotting to identify the presence of CYPs, antioxidants, and HPV proteins in Caski-derived EVs and to calculate the relative protein fold expression level of CYPs and AOEs in U1 cells upon treatment with CCS/CCS-EVs. We used the following primary antibodies: GAPDH Rabbit Mab, 1:2000 dilution (Cell Signaling Technology, Danvers, MA, USA), catalogue #2118; CYP1A1 rabbit Mab, 1:200 dilution (Proteintech Group, Inc., Rosemont, IL, USA), catalogue #13241-1-AP; CYP1B1 Rabbit Mab, 1:500 dilution (Santa Cruz Biotechnology, Dallas, TX, USA), catalogue #sc-32882; CYP2A6 Mouse Mab, 1:200 dilution (Abcam, Cambridge, MA, USA), catalogue #ab3570; SOD1 Mouse Mab, 1:1500 dilution, catalog #sc-101523; SOD2 Mouse Mab, 1:500 dilution, catalogue #sc-133254; Catalase Mouse Mab, 1:1200 dilution (Santa Cruz Biotechnology Inc., Dallas, TX, USA), catalog #21260-1-AP; PRDX6 Rabbit Mab, 1:500 dilution (LifeSpan Biosciences, Inc., Seattle, WA, USA), catalog #LS-C162131; CD63, Rabbit Pab, 1:200 dilution (Proteintech Group, Rosemont, IL, USA), catalog #25682-1-AP. CD81 rabbit Mab 1:400 dilution (Santa Cruz Biotechnology Inc., Dallas, TX, USA), catalog #sc-9158. While GAPDH was used as loading control for cellular proteins, CD63 and CD81 were used as loading controls for EV proteins.

### 2.11. EVs Uptake

The CCS-EVs were labeled with Green Fluorescent Protein (GFP) using Exo-GlowTM EVs Labeling Kits (System Biosciences, Palo Alto, CA, USA) as described previously [24]. EV pellet containing ~42 µg of protein in 500 µL of 1X PBS was incubated with 50 µL of 10X Exo-Green dye at 37 °C for 30 min. The labeling reaction was stopped by adding 100 µL of Exo Quick-TC, provided along with the kit. The labeled CCS-EVs were exposed to U1 cells for 6 h, and their uptake was monitored under fluorescent microscope and flow cytometry.

### 2.12. Statistical Analysis

Statistical analyses were performed using Graphpad Prism 7.0 (La Jolla, CA, USA). All data were presented as mean ± SEM of 3–5 independent experiments. Student’s t-test or one-way ANOVA were used to calculate the statistical differences between the control and the treated groups, where appropriate. Significant difference was considered at *p* < 0.05.

## 3. Results

### 3.1. Cell Culture Supernatant from Caski Cells Enhances Oxidative Stress and Viral Load in Differentiated U1 Cell Line

**Figure 1:**
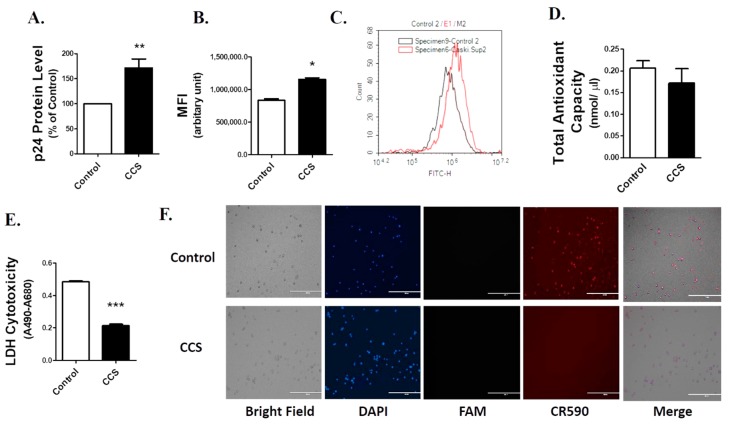
Caski cell culture supernatant (CCS) increases human immunodeficiency virus (HIV)-1 replication and oxidative stress in differentiated U1 cell line. (**A**) Differentiated U1 cell line was treated with 250 µL of CCS every 24 h for 4 days. p24 ELISA was performed on the supernatant obtained from the treatment to measure the viral load. To minimize high standard deviation of the mean due to variability in absorbance values in different experiments, we converted the control absorbance values to 100% and normalized the values of treated groups to % of the control. (**B**) Reactive oxygen species (ROS) was measured in differentiated U1 cell line after 4 days exposure of CCS. The treated cells were incubated with 2′,7′- dichlorodihydrofluorescein diacetate (H2DCFDA), the fluorescence of which was monitored at maximum excitation and emission spectra of 495 nm and 529 nm, respectively, using flow cytometry. (**C**) Shows the graphical representation of ROS increase in CCS-treated U1 cells (red graph) versus control cells (grey graph). X-axis represents mean fluorescence intensity (MFI), showing ROS level. (**D**) Total antioxidant capacity of the cells was measured in CCS-treated cells using the Total Antioxidant Capacity Assay Kit. The values on the Y-axis represents the total amount of reduced Cu^+^ in nmol/µL, which quantitatively gives the measure of antioxidant capacity of the cells. (**E)** Cytotoxicity after CCS exposure was measured using the Pierce^TM^ LDH cytotoxicity assay kit. The values on the Y-axis represent the absorbance values of formazan dye at 490 nm, which gives the measure of cytotoxicity. The mean absorbance is obtained by subtracting the background absorbance at 680 nm. All the data were obtained from the mean of at least three independent experiments with the error bars representing standard error of mean. Significant difference was considered at *p* < 0.05. *, **, *** represent *p* < 0.05, *p* < 0.005, and *p* < 0.0005, respectively. (**F**) Apoptotic DNA damage was examined using Apoptag^®^ Iso Dual Fluorescence Apoptosis Detection Kit. 4′,6-diamidino-2-phenylindole (DAPI), Fluorescein amidite (FAM), and CR590 dyes were used to stain the nucleus, DNase Type II and I of DNA breaks, respectively.

The exposure of cell culture supernatant (CCS) for 4 days significantly (*p*-value = 0.0084, *n* = 5) increased the viral load in HIV-1-infected macrophage cell lines (U1) by approximately 1.7-fold compared to the control (untreated cells) (Figure 1A). ROS acts as a secondary messenger for inducing HIV-1 replication in cells latently infected with HIV-1 [28]. To determine whether the viral replication is associated with oxidative stress, we measured the levels of ROS in the CCS-treated U1 cells. Our results showed that four days exposure of CCS induces ROS by ~1.25-fold (*p*-value = 0.0169, *n* = 3) in U1 cells (Figure 1B). Figure 1C shows a graphical representation of the ROS measurements shown in Figure 1B. During oxidative stress, cells employ antioxidant enzymes and proteins to neutralize the excess ROS, which may eventually wear away the total antioxidant capacity of the cells. Therefore, we monitored antioxidant capacity of U1 cells after four days of CCS treatment using the total antioxidant capacity (TAC) assay. Although not significant, the data presented in Figure 1D shows a trend of TAC decrease in CCS-treated cells compared to the control.

Next, we were interested to examine whether the excessive ROS induces cellular toxicity and DNA damage after CCS treatment. The cytotoxicity depicted in Figure 1E was performed using the LDH assay. Interestingly, the four-day treatment of CCS significantly (*p*-value < 0.0001, *n* = 6) decreased the cytotoxicity in U1 cells by ~60% (Figure 1E). To further confirm this result, we monitored apoptotic DNA fragmentation in CCS-treated U1 cells. Figure 1F shows the fluorescent images of control and CCS-treated U1 cells labeled with 4′,6-diamidino-2-phenylindole(DAPI), fluorescein amidite (FAM), and CR590 dyes, which were used to stain the nucleus, DNA fragmentation with DNase Type II, and Type I ends, respectively. DNA fragmentation with Type I ends, commonly observed in most cells, occurs within the nucleus, which is indicative of apoptosis by self-driven cell disassembly. In contrast, DNA fragmentation with Type II ends occurs in lysosomes of the phagocytes, where they eliminate the remains of apoptotic bodies [29]. The merged panel of Figure 1F shows reduced fluorescence intensity for CR590 in CCS-treated U1 cells compared to the control, which suggests that treatment of CCS protects U1 cells from DNase Type I of DNA break. We did not observe any signal with FAM dye in both the control and treated cells, which suggests the absence of DNase Type II of DNA break.

### 3.2. Cell Culture Supernatant from Caski Cells Induces CYP Expression in U1 Cells

**Figure 2:**
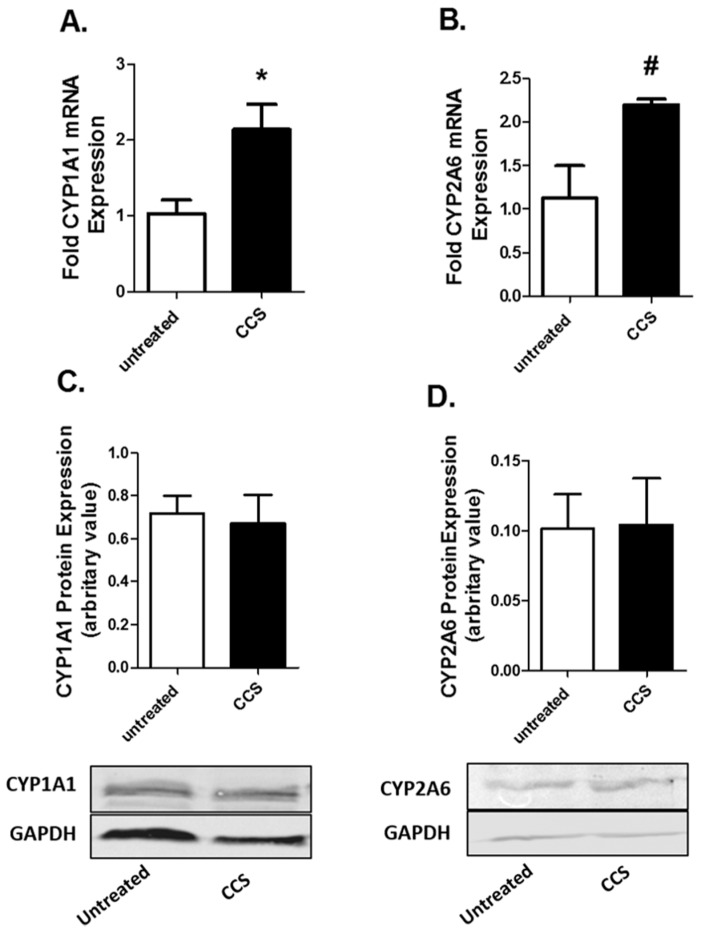
Caski cell culture supernatant (CCS) enhances cytochrome P450 (CYP) expression in differentiated U1 cell line. Differentiated U1 cell line was treated with 250 µL of CCS every 24 h for 4 days. Expression of CYP1A1 and CYP2A6 at the mRNA ((**A**) and (**B**)) and protein ((**C**) and (**D**)) level were monitored in the treated cells. Data were obtained from the mean of at least three independent experiments with the error bars representing the standard error of the mean. Significant difference (*) was considered at *p* < 0.05. * and # represent 0.1< *p* >0.05 respectively.

CYP enzymes are known to generate ROS as byproducts, while metabolizing various endogenous and exogenous substances within cells. Therefore, we monitored the mRNA and protein expression of CYPs (CYP 1A1 and 2A6) in U1 cells after four days of CCS exposure. We particularly examined the expressions of CYP 1A1 and 2A6, because these CYPs are significantly expressed in U1 cells, and they are induced by various xenobiotics including tobacco constituents and environmental contaminants via an oxidative stress pathway [30]. The four-day exposure of CCS significantly increased the expression of CYP1A1 and CYP2A6 at the mRNA level (Figure 2A,B) but not at the protein level (Figure 2C,D; Figure S1).

### 3.3. Cell Culture Supernatant from Caski Cells Have No Significant Effect on AOEs Expression in U1 Cells

**Figure 3:**
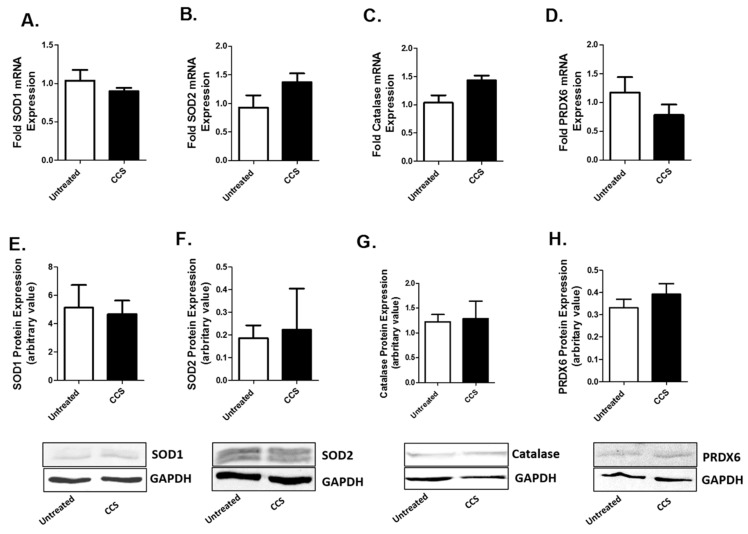
Caski cell culture supernatant (CCS) alters antioxidant enzyme (AOE) expression in differentiated U1 cell line. Differentiated U1 cell line was treated with 250 µL of CCS every 24 h for 4 days. Expressions of SOD1, SOD2, catalase, and PRDX6 were monitored at mRNA (**A–D**) and protein (**E–H**) levels in the treated cells. Data were obtained from the mean of at least three independent experiments with the error bars representing the standard error of the mean.

AOE expressions are expected to rise at the time of oxidative stress as a cellular response to combat the resulting oxidative damage. Therefore, we monitored the mRNA and protein expressions of major AOEs (SOD1, SOD2, catalase, and PRDX6) in U1 cells after four days of CCS exposure. The four-day exposure of CCS did not show a significant effect on the expression of the AOEs at protein as well as mRNA level (Figure 3; Figure S2).

### 3.4. Characterization of EVs derived from Caski Cells

**Figure 4:**
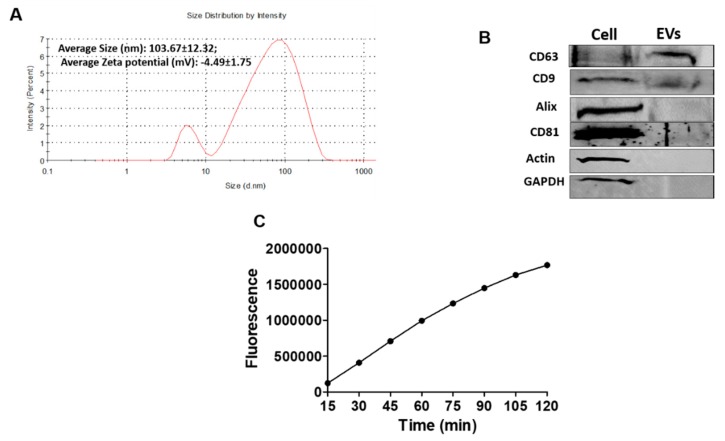
Characterization of extracellular vesicles (EVs) derived from Caski cells. (**A**) Representative plots from Zetasizer showing size distribution and zeta potential of Caski cell-derived EVs. (**B**) Protein obtained from Caski cells and their derived EVs was examined for the expression of EVs markers, CD63, CD9, Alix, and CD81. The housekeeping proteins of cells, actin, and glyceraldehyde 3-phosphate dehydrogenase (GAPDH), were also examined in both cells and their EVs. (**C**) The EVs were characterized for their functional identity using acetylcholine esterase activity.

**Figure 5:**
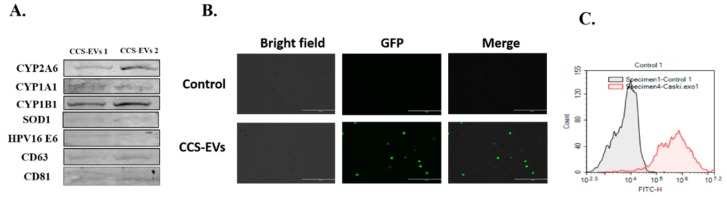
Differentiated U1 cell line uptake CCS-derived EVs (CCS-EVs) that contain oxidative stress factors. (**A**) The proteins obtained from CCS-derived EVs (CCS-EVs) were examined for the expression of EVs markers, CD63 and CD81, CYPs (1A1, 1B1, 2A6) and AOEs (SOD1, SOD2, and catalase) and HPV 16 oncoproteins (E6 and E7). (**B**) To monitor the EVs uptake by U1 cells, we labeled the CCS-EVs with GFP (Green Fluorescent Protein) and treated the labeled EVs to U1 cells. After 6 h of incubation, we monitored the fluorescent intensity of GFP under fluorescent microscope. (**C**) The GFP fluorescence was further quantified using flow cytometry.

Since EVs are one of the major factors that are secreted in the media from a variety of cells and are gaining attention in intercellular communication, we isolated EVs secreted from Caski cells. Recently, we reported isolation and characterizations of EVs from the media of monocytic cells [24], and in this study we essentially used similar techniques. The size and zeta potential of Caski cell-derived EVs were measured using Zetasizer (Figure 4A; Figure S3). The majority of EVs were ≤200 nm with an average size of 104 ± 12 nm and a zeta potential of –4.5 ± 1.8, which are indicative of substantially enriched EVs (Figure 4A). According to ISEV guidelines for extracellular vesicles characterization [31], the EVs were characterized using EV marker proteins CD63, CD9, Alix, and CD81, as well as acetylcholine esterase activity (Figure 4B,C). We measured the above-mentioned EV markers in both Caski cells as well as in their EVs. As expected, CD63 and CD9 were substantially packaged in their EVs (Figure 4B). However, the relative expression of Alix and CD81 was very low in EVs compared to that of Caski cells. As shown in Figure 5A, it is noted that Alix and CD81 are easily detectable when they are measured only in EVs, using a relatively high antibody concentration. Further, the commonly used housekeeping genes of cells, actin, and GAPDH, were detected only in Caski cells but not in their EVs. Finally, the EVs demonstrated acetylcholine esterase activity (Figure 4C), suggesting the isolation of functional EVs.

### 3.5. U1 Cells Uptake CCS-derived EVs Containing Oxidative Stress Factors

To determine whether oxidative stress plays a potential role in Caski cell-induced effects on U1 cells, we measured important oxidative stress-inducing CYP enzymes, common antioxidant enzymes (AOE), and HPV proteins in Caski-derived EVs. Our Western blot images showed the expression of CYP enzymes (CYP 1A1, 1B1, and 2A6), AOE (SOD1), and HPV 16 oncoprotein E6 in CCS-derived EVs (Figure 5A; Figure S4).

To show that U1 cells take up these EVs, we first labeled the EVs with GFP and monitored their uptake by U1 cells using fluorescent microscopy and flow cytometry after 6 h. The merged panel of Figure 5B shows higher GFP fluorescence in CCS-EVs treated U1 cells, compared to the untreated cells, indicating the EVs uptake by U1 cells. We further verified this result by measuring the GFP fluorescence using flow cytometry. In Figure 5C, we can see that the red graph (CCS-EVs treated U1 cells) shifts towards the far right compared to the grey graph (untreated U1 cells), indicating a higher mean fluorescence intensity for the former, and hence the uptake of CCS-EVs by U1 cells. Our observation is consistent with our previous study, where we observed the uptake of U937 monocyte-derived EVs by U1 cells in 6 h.

### 3.6. CCS-Derived EVs Enhance Oxidative Stress and Viral Load in U1 Cells

**Figure 6:**
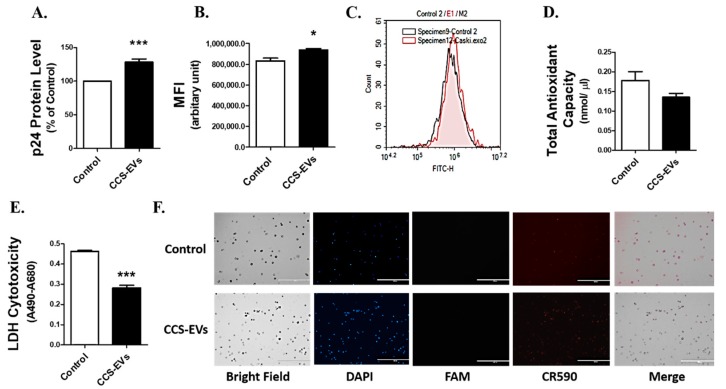
CCS-derived EVs (CCS-EVs) increase HIV-1 replication and oxidative stress in differentiated U1 cell line. (**A**) Differentiated U1 cell line was exposed with EVs derived from 1 mL of CCS (~42 µg of EVs protein) for 4 days. p24 ELISA was performed on the supernatant obtained from the treatment to measure the viral load. To minimize high standard deviation of the mean due to variability in absorbance values from different experiments, we converted the control absorbance values to 100% and normalized the values of treated groups to % of the control. (**B**) ROS was measured in differentiated U1 cell line after the 4 days exposure of CCS-EVs. The treated cells were incubated with H2DCFDA, and the fluorescence was monitored at maximum excitation and emission spectra of 495 nm and 529 nm, respectively, using flow cytometry. (**C**) Shows the graphical representation of ROS increase in CCS-EVs-treated U1 cells (red graph) versus control cells (grey graph). The X-axis represents mean fluorescence intensity (MFI), showing ROS level. (**D**) Total antioxidant capacity of the cells was measured in CCS-treated cells using the Total Antioxidant Capacity Assay Kit. The values on the Y-axis represent the total amount of reduced Cu+ in nmol/µL, which quantitatively gives the measure of antioxidant capacity of the cells. (**E**) Cytotoxicity after CCS exposure was measured using the Pierce^TM^ LDH Cytotoxicity Assay Kit. The values on the Y-axis represent the absorbance values of formazan dye at 490 nm, which give the measure of cytotoxicity. The mean absorbance is obtained by subtracting the background absorbance at 680 nm. All the data were obtained from the mean of at least three independent experiments with the error bars representing the standard error of mean. Significant differences * and *** represent *p* < 0.05 and *p* < 0.0005, respectively. (**F**) Apoptotic DNA damage was examined using Apoptag^®^ Iso Dual Fluorescence Apoptosis Detection kit. DAPI, FAM, and CR590 dyes were used to stain the nucleus, DNase Type II and I DNA breaks, respectively.

To examine if the Caski-derived EVs present in CCS were responsible for the increase in viral load in U1 cells, we treated the CCS-EVs to the U1 cells. U1 cells were treated with EVs isolated from 1 mL of CCS (equivalent to ~42 µg protein) and cultured for four days. Exposure of U1 cells to CCS-EVs for four days significantly (*p*-value = 0.0007, *n* = 6) increased the viral load by approximately 1.3-fold (Figure 6A). CCS-EVs treatment significantly (*p*-value = 0.0236, *n* = 3) increased the ROS level in U1 cells (Figure 6B,C). Although the data were not statistically significant, we observed a decreasing trend in the total antioxidant capacity of the U1 cells after CCS-EVs treatment (Figure 6D). The increase in ROS and decrease in total antioxidant capacity both are indicative of an overall increase in oxidative stress. Next, we monitored the cytotoxicity and DNA damage in CCS-EVs treated U1 cells. Interestingly, we observed ~40% decrease (*p*-value < 0.0001, *n* = 6) in cytotoxicity (Figure 6E) in the CCS-EVs treated U1 cells. Similarly, the merged panel of Figure 6F shows lower CR590 fluorescence for the treated cells than for the control, indicating a lower extent of DNA damage in the treated cells. These results are in agreement with the results obtained from CCS-treated U1 cells.

### 3.7. Treatment of Antioxidants, CYP-inhibitors, and Chemodietary Agents Reduce Viral Load in Caski EVs-Treated U1 Cells

**Figure 7:**
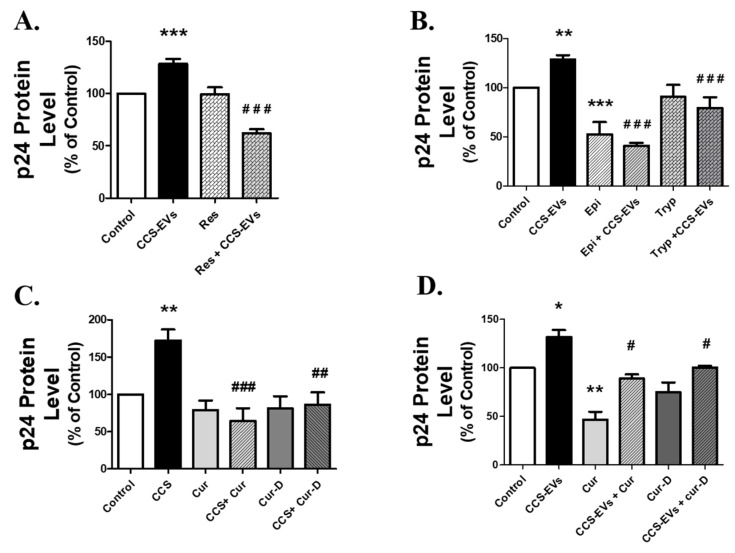
Treatment of antioxidant, CYP inhibitors, and chemodietary agents reduce CCS or CCS-derived EVs (CCS-EVs)-induced HIV-1 replication in differentiated U1 cell line. To confirm the role of CYP-induced oxidative stress in CCS-EVs treated U1 cells, we treated differentiated U1 cell line with the antioxidant, resveratrol (**A**. Res, 25 µM), CYP1A1 inhibitor, ellipticine (**B**. Epi 1 µM), and CYP2A6 inhibitor, tryptamine (**B**. Tryp, 20 µM). We also treated the U1 cells exposed to CCS (**C**) or CCS-EVs (**D**) with curcumin (cur, 20 µM) and cucurbitacin-D (Cur-D, 0.1 µM). All the data were obtained from the mean of at least three independent experiments with the error bars representing the standard error of mean. Significant differences *, **, *** represent *p* < 0.05, *p* < 0.005, and *p* < 0.0005, respectively, when compared to the control. #, ##, ### represent *p* < 0.05, *p* < 0.005, and *p* < 0.0005, respectively, when compared to CCS or CCS-EVs groups.

To confirm that HIV-1 replication is occurring via a CYP-mediated oxidative stress pathway, we treated the CCS-EVs exposed U1 cells with an antioxidant (resveratrol) CYP1A1-selective inhibitor (ellipticine), and CYP2A6-selective inhibitor (tryptamine). The U1 cells were exposed to EVs isolated from 1 mL of CCS (comprising of ~42 μg of EVs protein) for four days. Then, resveratrol (25 µM), ellipticine (Epi, 1 µM), and tryptamine (Tryp, 20 µM) were added to the CCS-EVs treated U1 cells every 24 h for four days. Treatment of resveratrol (*p*-value < 0.0001, *n* =3), ellipticine (*p*-value < 0.0001, *n* =3), or tryptamine (*p*-value < 0.0001, *n* =3) significantly reduced the viral load in CCS-EVs exposed U1 cells, suggesting the role of CYP 1A1 and 2A6, and oxidative stress on viral load increase in U1 cells (Figure 7A,B).

Chemodietary agents such as curcumin and cucurbitacin-D are known to reduce cancer progression [32,33], as well as HIV-1 replication [34,35], through their antioxidant potential. We treated curcumin (20 µM) and cucurbitacin-D (0.1 µM) to CCS or CCS-EVs-treated U1 cells for four days, every 24 h, with an assumption that they would combat the oxidative stress caused by CCS or CCS-EVs and hence reduce the viral replication in U1 cells. As expected, treatment of curcumin (*p*-value < 0.0001, *n* = 7) and cucurbitacin-D (*p*-value < 0.001, *n*= 5) significantly reduced the viral load in CCS-treated cells (Figure 7C). We also observed a significant decrease in viral load in CCS-EVs-treated U1 cells with curcumin (*p*-value < 0.01, *n* = 3) or cucurbitacin-D (*p*-value < 0.001, *n* = 3) treatment (Figure 7D).

## 4. Discussion

**Figure 8:**
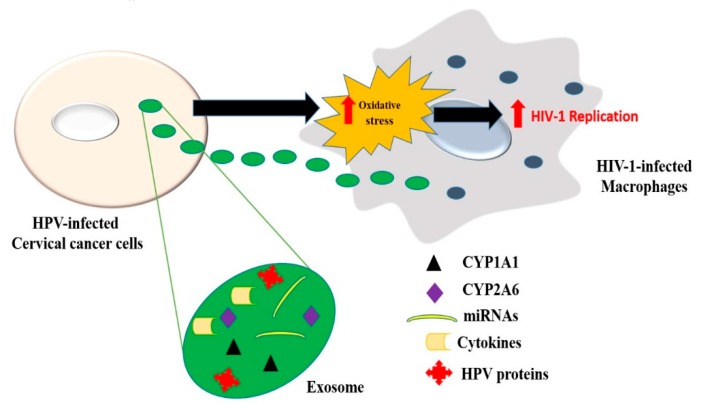
Proposed model for HPV–HIV-1 interaction via EVs. EVs from cervical cancer cells carry oxidative stress factors that exacerbate HIV-1 replication in HIV-1-infected macrophages. As a result of constant oxidative stress, cervical cancer cells package oxidative stress factors like CYP enzymes (CYP 1A1, 2A6, 1B1) and HPV oncoproteins, as well as potentially miRNAs and cytokines, into EVs and transfer them to cells (e.g., macrophages at distant sites). The EVs then release the oxidative stress factors in recipient cells, which further enhance ROS production in the cells. The ROS thus generated provides as a secondary messenger to trigger HIV-1 replication in macrophages.

There is a high risk and prevalence of HPV co-infection in HIV-1-infected individuals, which cause severe forms of cervical cancer. HIV-1 modulates the host cell microenvironment for HPV invasion, through destruction of tight junctions between the cells and immunosuppression [4]. However, whether cross-talk between HPV and HIV-1-infected cells exists, is unknown. The current study highlights EVs as a means of intercellular communication between the two cells. Here, we demonstrate for the first time that HPV-infected cervical cancer cells transfer oxidative stress factors (such as CYPs and HPV proteins) via EVs to the recipient differentiated U1 cell line and induce HIV-1 replication. The EV CYPs (CYP 1A1 and 2A6) exert an additive effect on the basal CYPs of the recipient cells. The cumulative CYPs promote the metabolism of endogenous and exogenous substances (environmental contaminants), thereby generating ROS, which eventually exacerbates HIV-1 replication in the recipient cells (Figure 8). Our study also demonstrates the role of specific antioxidants in attenuating oxidative stress-induced HIV-1 replication.

In the current study, we show the interaction between HPV and HIV-1 infected cells using Caski cells and U1 cells. Caski and U1 are both validated cell lines (ATCC) and are widely used for mechanistic studies [36,37]. The data from these cell lines were also validated using primary infected cells and tissues, which show similar effects [24,38]. Therefore, it is logical to use these cell lines for studying the potential interactions between HPV and HIV-1 via EVs. To determine the interaction between Caski cells and U1 cells, we initially exposed the U1 cells with Caski cell culture supernatant (CCS). Exposure of CCS to U1 cells significantly increased oxidative stress and HIV-1 replication in U1 cells. Initially, we assumed that Caski cells released oxidative stress factors into the cell culture media, which when transferred to the U1 cells, and induced oxidative stress and viral replication in U1 cells. Later, we were interested to investigate if Caski cells communicate with U1 cells via EVs in the presence of CCS exposure. Therefore, we isolated EVs from CCS-treated cells and exposed them to U1 cells. Interestingly, exposure of CCS-derived EVs (CCS-EVs) also revealed higher viral load and ROS level.

We recently demonstrated that oxidative stress, generated via CYP-mediated metabolism of tobacco constituents, triggers HIV-1 replication in monocytes and macrophages [23,24]. We showed that ROS thus generated, induces translocation of cytosolic NF-κB subunits into the nucleus, where it binds to the core enhancer region of the HIV-1 long terminal repeat (LTR) to induce HIV-1 transcription. ROS generated through CCS/CCS-derived EVs induces HIV-1 replication in U1 cells via a similar mechanism. Furthermore, a redox state of the monocytes/macrophages is also reported to regulate the expression of C-C chemokine receptor type 5 (CCR5) and C-X-C chemokine receptor type 4 (CXCR4), which are major coreceptors for HIV-1 entry into monocytes/macrophages [39,40]. ROS elevation through CCS/CCS-derived EVs facilitates the viral entry and hence increases the viral load within the U1 cells via overexpression of these chemokine receptors. Treatment of antioxidants such as glutathione, glutathione ester, and N-acetyl-L-cysteine was reported to suppress HIV-1 expression in U1 cells, which strongly supports the involvement of oxidative stress in HIV-1 expression in macrophages [41]. Our findings are consistent with previous reports, and they provide evidence for CCS/CCS-derived EVs as a novel source of ROS for HIV-1 replication in macrophages.

We observed that exposure of CCS/CCS-derived EVs decreased cell death and DNA damage in U1 cells, despite increasing oxidative stress. Under stress conditions, cells initially attempt to protect themselves from the insult by activating signaling pathways that promote cell survival. If they are unable to overcome the insult, they activate death signaling pathways [42]. In the current context, treatment with CCS/CCS-derived EVs is likely to activate cell survival pathways, such as antioxidant defense system, heat shock and unfolded protein response, and DNA damage repair, to rescue U1 cells from oxidative insult [42]. Furthermore, most cancer cells, including cervical cancer cells, are known to overexpress and release anti-apoptotic factors like survivin, which arrest apoptosis by inhibiting caspase activation [43,44]. It could be possible that the protective mechanism is mediated by anti-apoptotic factors like survivin in the group treated with CCS-derived EVs. In addition, HIV-1 protein Nef, is known to protect HIV-1-infected host cells by inhibiting apoptosis signal-regulating kinase 1 (ASK1), thus preventing Fas and TNF-α-mediated apoptotic cell death [45]. HIV-1 could be promoting cell survival in the treated U1 cells, to frame a favorable environment for its multiplication.

Next, we were interested in finding which oxidative stress factors are present in CCS/CCS-derived EVs and which mechanistic pathways they trigger to induce oxidative stress and consequently HIV-1 replication in U1 cells. Overexpression of CYPs is observed in several cancers, including cervical cancer [46], and CYPs are known to generate excessive ROS as a byproduct during the phase-I metabolism of various endogenous and exogenous substrates [47]. Taking these facts into consideration, we examined the effect of CCS on the expression of CYPs in U1 cells. We particularly examined the expression of CYP1A1 and CYP2A6, because these enzymes are expressed in monocytic cells. CYP1A1 and CYP2A6 are the major enzymes for metabolizing polyaryl hydrocarbons and nicotine present in cigarette smoke, respectively, as well as environmental contaminants [48,49]. The concurrent upregulation of CYPs and elevation in ROS levels suggest that the oxidative stress in U1 cells could occur via a CYP-mediated pathway. We did not observe any change in the expression of antioxidant enzymes after CCS exposure. However, there was a decrease in the total antioxidant capacity of the cells after CCS or CCS-mediated EVs treatment.

In this study, we observed an increase in the expression of CYPs at the mRNA level but not at the protein level. We observed this kind of discrepancy in our previous studies as well [21]. As mRNA is translated into protein, it is expected that there is a correlation between the expression of genes at the mRNA and the protein level. However, it is not always necessary that mRNA expression changes reflect similar changes in protein expression [50,51,52]. The reason for a decrease in protein expression, despite increased mRNA expression, could be due to various transcriptional and post-translational modifications, differential stability of mRNAs versus proteins, or due to interference by different miRNAs [53,54]. This is consistently the case for CYP1A1 expression upon exposure to cigarette smoke condensate and benzo(a)pyrene [23,48].

Next, we verified the presence of CYPs 1A1, 2A6, and 1B1 in CCS-derived EVs, which further strengthens our assumption about the involvement of a CYP-pathway in CCS-induced oxidative stress. To further confirm the role of CYPs in oxidative stress-induced HIV-1 replication, we treated the U1 cells exposed to CCS-derived EVs with antioxidants and CYP inhibitors, which significantly reduced the viral load in the treated U1 cells. Overall, our findings suggest that Caski cells transfer CYPs to U1 cells via EVs. Upon reaching the U1 cells, these EVs release CYPs into the cytosol, where they induce oxidative stress and subsequently HIV-1 replication. Our observations are in agreement with our previous reports, where we observed a higher expression of CYPs, oxidative stress, and HIV-1 viral load in the plasma samples of HIV-1 smokers, which shows the association of a CYP-mediated oxidative stress pathway in HIV-1 replication [55]. Recently, we confirmed this association in vitro in U1 cells, in which we demonstrated that CYP1A1 metabolizes benzo(a)pyrene (a harmful carcinogen in cigarette smoke) and causes increased production of ROS, which subsequently triggers HIV-1 replication in the U1 cells [23].

Apart from CYPs, we also observed the presence of other oxidative stress-inducing factors, such as HPV type 16 oncoprotein E6, in the CCS-derived EVs. HPV16 E6 and E7 oncoproteins are known to cause chronic oxidative stress in HPV-infected and uninfected cells via NOX2 activation [14]. In addition, CCS-derived EVs may also contain various cytokines [56] and miRNAs that may trigger HIV-1 replication in differentiated U1 cell line. Cytokines, such as TNF-α, trigger HIV-1 gene transcription through activation of NF-κB; IL-6 increases the expression of viral proteins and RT; IL-6, together with TNF-α, have a synergistic effect on HIV-1 replication [57]. Micro RNA (miRNA) 34a and miRNA 181 are reported to enhance HIV-1 viral load by inhibiting cellular restriction factors (e.g., p21, TASK, and SAMHD1), which inhibit different stages of the HIV-1 life cycle [58,59]. Since these miRNAs are also expressed in cervical cancer cells, it is possible that they are being transported to host cells via EVs, where they contribute to HIV-1 replication.

Curcumin and cucurbitacin-D are shown to inhibit cancer growth in the cervix by inducing apoptosis and arresting the cell cycle [32,33]. In addition to their anti-cancer properties, curcumin and cucurbitacin also suppress HIV-1 pathogenesis through their antioxidant potential [34,35]. Curcumin is shown to reduce HIV-1 transcription by inhibiting the HIV-1 protein, Tat-mediated LTR promotor transactivation [34]. Considering their potential antioxidant and antiviral properties, we examined the preventive effect of these agents to HIV-1 replication induced by CCS/CCS-derived EVs. As expected, treatment of both the compounds significantly reduced HIV-1 viral load induced by CCS or CCS-derived EVs in U1 cells. Curcumin and cucurbitacin-D are known to scavenge hydroxyl and superoxide ions at higher concentrations [60]. It is therefore possible that these compounds inhibit viral replication by lowering the ROS level. Furthermore, the use of curcumin and cucurbitacin-D as antioxidants to block viral replication is clinically safer, as these natural antioxidants are less carcinogenic compared to synthetic antioxidants, such as butylated hydroxytoluene (BHT) and butylated hydroxyanisole (BHA). Furthermore, the use of these chemodietary agents in HIV-1 patients with cervical cancer is more beneficial because of their dual effect on reducing cancer progression and HIV-1 replication.

## 5. Conclusions

In conclusion, increased oxidative stress is associated with the effect of CCS-EVs on increasing HIV replication in differentiated U1 cell line. We also show that the viral replication undergoes a CYP-mediated oxidative stress pathway, and viral replication is reduced with treatment of chemodietary agents like curcumin and cucurbitacin-D. The present study, therefore, provides important information regarding biological interactions between HPV and HIV via EVs leading to enhanced HIV-1 replication. This study also provides a rationale to examine the role of EVs, CYP and oxidative stress pathways in HPV–HIV interactions in tobacco users, because the prevalence of cigarette smoking in both HIV- and HPV-infected populations is higher than in the general population [61,62].

## Supplementary Materials

The following are available online at https://www.mdpi.com/1999-4915/12/2/239/s1, Figure S1: Original Western data of Caski cells culture-mediated CYP expression in U1 cells, Figure S2: Original Western data of Caski cells culture-mediated AOE expression in U1 cells, Figure S3: Original Western data of Caski-derived EVs marker proteins, Figure S4: Original Western data of Caski cell culture-induced EVs proteins.

## References

[1] Centers for Disease Control and Prevention Cancers Associated with Human Papillomavirus, United States—2012–2016. https://www.cdc.gov/cancer/hpv/statistics/cases.htm.

[2] Clifford G.M., Tully S., Franceschi S. (2017). Carcinogenicity of Human Papillomavirus (HPV) Types in HIV-Positive Women: A Meta-Analysis from HPV Infection to Cervical Cancer. Clin. Infect. Dis..

[3] Stuardo V., Agusti C., Godinez J.M., Montoliu A., Torne A., Tarrats A., Alcalde C., Martin D., Fernandez-Montoli E., Vanrell C. (2012). Human papillomavirus infection in HIV-1 infected women in Catalonia (Spain): Implications for prevention of cervical cancer. PLoS ONE.

[4] Brickman C., Palefsky J.M. (2015). Human papillomavirus in the HIV-infected host: Epidemiology and pathogenesis in the antiretroviral era. Curr. HIV/AIDS Rep..

[5] Maiman M., Fruchter R.G., Clark M., Arrastia C.D., Matthews R., Gates E.J. (1997). Cervical cancer as an AIDS-defining illness. Obstet. Gynecol..

[6] Manju V., Balasubramanian V., Nalini N. (2002). Oxidative stress and tumor markers in cervical cancer patients. J. Biochem. Mol. Biol. Biophys..

[7] Filippova M., Filippov V., Williams V.M., Zhang K., Kokoza A., Bashkirova S., Duerksen-Hughes P. (2014). Cellular levels of oxidative stress affect the response of cervical cancer cells to chemotherapeutic agents. Biomed. Res. Int..

[8] De Marco F. (2013). Oxidative stress and HPV carcinogenesis. Viruses.

[9] Naidu M.S., Suryakar A.N., Swami S.C., Katkam R.V., Kumbar K.M. (2007). Oxidative stress and antioxidant status in cervical cancer patients. Indian J. Clin. Biochem..

[10] Manju V., Kalaivani Sailaja J., Nalini N. (2002). Circulating lipid peroxidation and antioxidant status in cervical cancer patients: A case-control study. Clin. Biochem..

[11] Goncalves T.L., Erthal F., Corte C.L., Muller L.G., Piovezan C.M., Nogueira C.W., Rocha J.B. (2005). Involvement of oxidative stress in the pre-malignant and malignant states of cervical cancer in women. Clin. Biochem..

[12] Yim E.K., Park J.S. (2005). The role of HPV E6 and E7 oncoproteins in HPV-associated cervical carcinogenesis. Cancer Res. Treat..

[13] Ding B., Chi S.G., Kim S.H., Kang S., Cho J.H., Kim D.S., Cho N.H. (2007). Role of p53 in antioxidant defense of HPV-positive cervical carcinoma cells following H2O2 exposure. J. Cell. Sci..

[14] Marullo R., Werner E., Zhang H., Chen G.Z., Shin D.M., Doetsch P.W. (2015). HPV16 E6 and E7 proteins induce a chronic oxidative stress response via NOX2 that causes genomic instability and increased susceptibility to DNA damage in head and neck cancer cells. Carcinogenesis.

[15] Liu J., Sun H., Wang X., Yu Q., Li S., Yu X., Gong W. (2014). Increased exosomal microRNA-21 and microRNA-146a levels in the cervicovaginal lavage specimens of patients with cervical cancer. Int. J. Mol. Sci..

[16] Honegger A., Leitz J., Bulkescher J., Hoppe-Seyler K., Hoppe-Seyler F. (2013). Silencing of human papillomavirus (HPV) E6/E7 oncogene expression affects both the contents and the amounts of extracellular microvesicles released from HPV-positive cancer cells. Int. J. Cancer.

[17] Théry C., Zitvogel L., Amigorena S. (2002). Exosomes: Composition, biogenesis and function. Nat. Rev. Immunol..

[18] Tkach M., Thery C. (2016). Communication by Extracellular Vesicles: Where We Are and Where We Need to Go. Cell.

[19] Harden M.E., Munger K. (2017). Human papillomavirus 16 E6 and E7 oncoprotein expression alters microRNA expression in extracellular vesicles. Virology.

[20] Khan S., Aspe J.R., Asumen M.G., Almaguel F., Odumosu O., Acevedo-Martinez S., De Leon M., Langridge W.H., Wall N.R. (2009). Extracellular, cell-permeable survivin inhibits apoptosis while promoting proliferative and metastatic potential. Br. J. Cancer.

[21] Rao P., Ande A., Sinha N., Kumar A., Kumar S. (2016). Effects of Cigarette Smoke Condensate on Oxidative Stress, Apoptotic Cell Death, and HIV Replication in Human Monocytic Cells. PLoS ONE.

[22] Ivanov A.V., Valuev-Elliston V.T., Ivanova O.N., Kochetkov S.N., Starodubova E.S., Bartosch B., Isaguliants M.G. (2016). Oxidative Stress during HIV Infection: Mechanisms and Consequences. Oxid. Med. Cell. Longev..

[23] Ranjit S., Sinha N., Kodidela S., Kumar S. (2018). Benzo(a)pyrene in Cigarette Smoke Enhances HIV-1 Replication through NF-κB Activation via CYP-Mediated Oxidative Stress Pathway. Sci. Rep..

[24] Haque S., Sinha N., Ranjit S., Midde N.M., Kashanchi F., Kumar S. (2017). Monocyte-derived exosomes upon exposure to cigarette smoke condensate alter their characteristics and show protective effect against cytotoxicity and HIV-1 replication. Sci. Rep..

[25] Tang Y.T., Huang Y.Y., Zheng L., Qin S.H., Xu X.P., An T.X., Xu Y., Wu Y.S., Hu X.M., Ping B.H. (2017). Comparison of isolation methods of exosomes and exosomal RNA from cell culture medium and serum. Int. J. Mol. Med..

[26] Konoshenko M.Y., Lekchnov E.A., Vlassov A.V., Laktionov P.P. (2018). Isolation of Extracellular Vesicles: General Methodologies and Latest Trends. Biomed. Res. Int..

[27] Kodidela S., Wang Y., Patters B.J., Gong Y., Sinha N., Ranjit S., Gerth K., Haque S., Cory T., McArthur C. (2019). Proteomic Profiling of Exosomes Derived from Plasma of HIV-Infected Alcohol Drinkers and Cigarette Smokers. J. Neuroimmune Pharmacol..

[28] Pyo C.W., Yang Y.L., Yoo N.K., Choi S.Y. (2008). Reactive oxygen species activate HIV long terminal repeat via post-translational control of NF-kappaB. Biochem. Biophys. Res. Commun..

[29] Didenko V.V. (2011). 5’OH DNA breaks in apoptosis and their labeling by topoisomerase-based approach. Methods Mol. Biol..

[30] Ioannides C., Lewis D.F. (2004). Cytochromes P450 in the bioactivation of chemicals. Curr. Top. Med. Chem..

[31] Théry C., Witwer K.W., Aikawa E., Alcaraz M.J., Anderson J.D., Andriantsitohaina R., Antoniou A., Arab T., Archer F., Atkin-Smith G.K. (2018). Minimal information for studies of extracellular vesicles 2018 (MISEV2018): A position statement of the International Society for Extracellular Vesicles and update of the MISEV2014 guidelines. J. Extracell. Ves..

[32] Zaman M.S., Chauhan N., Yallapu M.M., Gara R.K., Maher D.M., Kumari S., Sikander M., Khan S., Zafar N., Jaggi M. (2016). Curcumin Nanoformulation for Cervical Cancer Treatment. Sci. Rep..

[33] Yallapu M.M., Jaggi M., Chauhan S.C. (2012). Curcumin nanoformulations: A future nanomedicine for cancer. Drug Discov. Today.

[34] Ali A., Banerjea A.C. (2016). Curcumin inhibits HIV-1 by promoting Tat protein degradation. Sci. Rep..

[35] Kumari N., Kulkarni A.A., Lin X., McLean C., Ammosova T., Ivanov A., Hipolito M., Nekhai S., Nwulia E. (2015). Inhibition of HIV-1 by curcumin A, a novel curcumin analog. Drug Des. Dev. Ther..

[36] Kim C.J., Um S.J., Kim T.Y., Kim E.J., Park T.C., Kim S.J., Namkoong S.E., Park J.S. (2000). Regulation of cell growth and HPV genes by exogenous estrogen in cervical cancer cells. Int. J. Gynecol. Cancer.

[37] Cottage A., Dowen S., Roberts I., Pett M., Coleman N., Stanley M. (2001). Early genetic events in HPV immortalised keratinocytes. Genes Chromosomes Cancer.

[38] Wang X., Tang S., Le S.Y., Lu R., Rader J.S., Meyers C., Zheng Z.M. (2008). Aberrant expression of oncogenic and tumor-suppressive microRNAs in cervical cancer is required for cancer cell growth. PLoS ONE.

[39] Saccani A., Saccani S., Orlando S., Sironi M., Bernasconi S., Ghezzi P., Mantovani A., Sica A. (2000). Redox regulation of chemokine receptor expression. Proc. Natl. Acad. Sci. USA.

[40] Tuttle D.L., Harrison J.K., Anders C., Sleasman J.W., Goodenow M.M. (1998). Expression of CCR5 increases during monocyte differentiation and directly mediates macrophage susceptibility to infection by human immunodeficiency virus type 1. J. Virol..

[41] Kalebic T., Kinter A., Poli G., Anderson M.E., Meister A., Fauci A.S. (1991). Suppression of human immunodeficiency virus expression in chronically infected monocytic cells by glutathione, glutathione ester, and N-acetylcysteine. Proc. Natl. Acad. Sci. USA.

[42] Fulda S., Gorman A.M., Hori O., Samali A. (2010). Cellular stress responses: Cell survival and cell death. Int. J. Cell. Biol..

[43] Xue Y., An R., Zhang D., Zhao J., Wang X., Yang L., He D. (2011). Detection of survivin expression in cervical cancer cells using molecular beacon imaging: New strategy for the diagnosis of cervical cancer. Eur. J. Obstet. Gynecol. Reprod. Biol..

[44] Khan S., Jutzy J.M., Aspe J.R., McGregor D.W., Neidigh J.W., Wall N.R. (2011). Survivin is released from cancer cells via exosomes. Apoptosis.

[45] Geleziunas R., Xu W., Takeda K., Ichijo H., Greene W.C. (2001). HIV-1 Nef inhibits ASK1-dependent death signalling providing a potential mechanism for protecting the infected host cell. Nature.

[46] Piotrowska H., Kucinska M., Murias M. (2013). Expression of CYP1A1, CYP1B1 and MnSOD in a panel of human cancer cell lines. Mol. Cell. Biochem..

[47] Zangar R.C., Davydov D.R., Verma S. (2004). Mechanisms that regulate production of reactive oxygen species by cytochrome P450. Toxicol. Appl. Pharmacol..

[48] Ranjit S., Midde N.M., Sinha N., Patters B.J., Rahman M.A., Cory T.J., Rao P.S., Kumar S. (2016). Effect of Polyaryl Hydrocarbons on Cytotoxicity in Monocytic Cells: Potential Role of Cytochromes P450 and Oxidative Stress Pathways. PLoS ONE.

[49] Earla R., Ande A., McArthur C., Kumar A., Kumar S. (2014). Enhanced nicotine metabolism in HIV-1-positive smokers compared with HIV-negative smokers: Simultaneous determination of nicotine and its four metabolites in their plasma using a simple and sensitive electrospray ionization liquid chromatography-tandem mass spectrometry technique. Drug Metab. Dispos..

[50] Shebl F.M., Pinto L.A., Garcia-Pineres A., Lempicki R., Williams M., Harro C., Hildesheim A. (2010). Comparison of mRNA and protein measures of cytokines following vaccination with human papillomavirus-16 L1 virus-like particles. Cancer Epidemiol. Biomarkers Prev..

[51] Mehra A., Lee K.H., Hatzimanikatis V. (2003). Insights into the relation between mRNA and protein expression patterns: I. Theoretical considerations. Biotechnol. Bioeng..

[52] Sarro S.M., Unruh T.L., Zuccolo J., Sanyal R., Luider J.M., Auer-Grzesiak I.A., Mansoor A., Deans J.P. (2010). Quantification of CD20 mRNA and protein levels in chronic lymphocytic leukemia suggests a post-transcriptional defect. Leuk. Res..

[53] Vogel C., Marcotte E.M. (2012). Insights into the regulation of protein abundance from proteomic and transcriptomic analyses. Nat. Rev. Genet..

[54] Greenbaum D., Colangelo C., Williams K., Gerstein M. (2003). Comparing protein abundance and mRNA expression levels on a genomic scale. Genome Biol..

[55] Ande A., McArthur C., Ayuk L., Awasom C., Achu P.N., Njinda A., Sinha N., Rao P.S., Agudelo M., Nookala A.R. (2015). Effect of mild-to-moderate smoking on viral load, cytokines, oxidative stress, and cytochrome P450 enzymes in HIV-infected individuals. PLoS ONE.

[56] Kodidela S., Ranjit S., Sinha N., McArthur C., Kumar A., Kumar S. (2018). Cytokine profiling of exosomes derived from the plasma of HIV-infected alcohol drinkers and cigarette smokers. PLoS ONE.

[57] Poli G., Bressler P., Kinter A., Duh E., Timmer W.C., Rabson A., Justement J.S., Stanley S., Fauci A.S. (1990). Interleukin 6 induces human immunodeficiency virus expression in infected monocytic cells alone and in synergy with tumor necrosis factor alpha by transcriptional and post-transcriptional mechanisms. J. Exp. Med..

[58] Farberov L., Herzig E., Modai S., Isakov O., Hizi A., Shomron N. (2015). MicroRNA-mediated regulation of p21 and TASK1 cellular restriction factors enhances HIV-1 infection. J. Cell. Sci..

[59] Jin C., Peng X., Liu F., Cheng L., Lu X., Yao H., Wu H., Wu N. (2014). MicroRNA-181 expression regulates specific post-transcriptional level of SAMHD1 expression in vitro. Biochem. Biophys. Res. Commun..

[60] Kunchandy E., Rao M.N.A. (1990). Oxygen radical scavenging activity of curcumin. Int. J. Pharm..

[61] Mzarico E., Gomez-Roig M.D., Guirado L., Lorente N., Gonzalez-Bosquet E. (2015). Relationship between smoking, HPV infection, and risk of Cervical cancer. Eur. J. Gynaecol. Oncol..

[62] Mdege N.D., Shah S., Ayo-Yusuf O.A., Hakim J., Siddiqi K. (2017). Tobacco use among people living with HIV: Analysis of data from Demographic and Health Surveys from 28 low-income and middle-income countries. Lancet Glob. Health.

