# Supplementary material for: Extracellular Vesicles from Human Papilloma Virus-Infected Cervical Cancer Cells Enhance HIV-1 Replication in Differentiated U1 Cell Line
Source: Viruses. 2020 Feb 21;12(2):239. doi: 10.3390/v12020239 (PMC7077309; doi:10.3390/v12020239)

Extracellular vesicles from human papilloma virus-infected cervical cancer cells enhance HIV-1 replication in macrophages

Sabina Ranjit[1], Sunitha Kodidela[1], Namita Sinha[1], Subhash Chauhan[1] and Santosh Kumar[1*]

[1]Department of Pharmaceutical Sciences, University of Tennessee Health Science Center, Memphis, TN 38163.

[*] Corresponding author

Santosh Kumar, Ph.D.

Professor and Assistant Dean

Department of Pharmaceutical Sciences

College of Pharmacy

University of Tennessee Health Science Center

881 Madison Ave, Room 456

Memphis, TN 38163

Email: ksantosh@uthsc.edu

Figure S1: Original Western data of Caski cells culture-mediated CYP expression in U1 cells

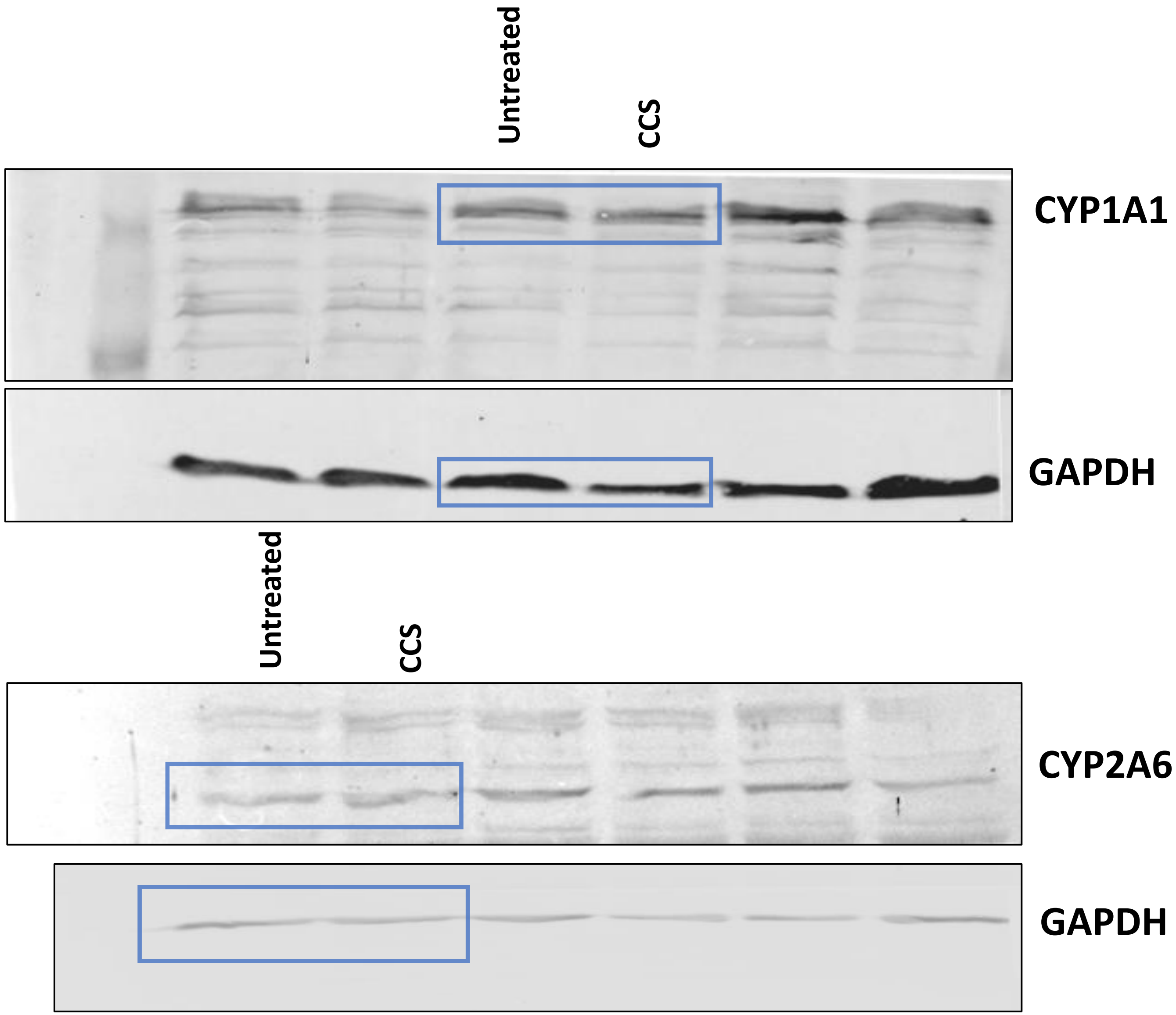

Figure S2: Original Western data of Caski cells culture-mediated AOE expression in U1 cells

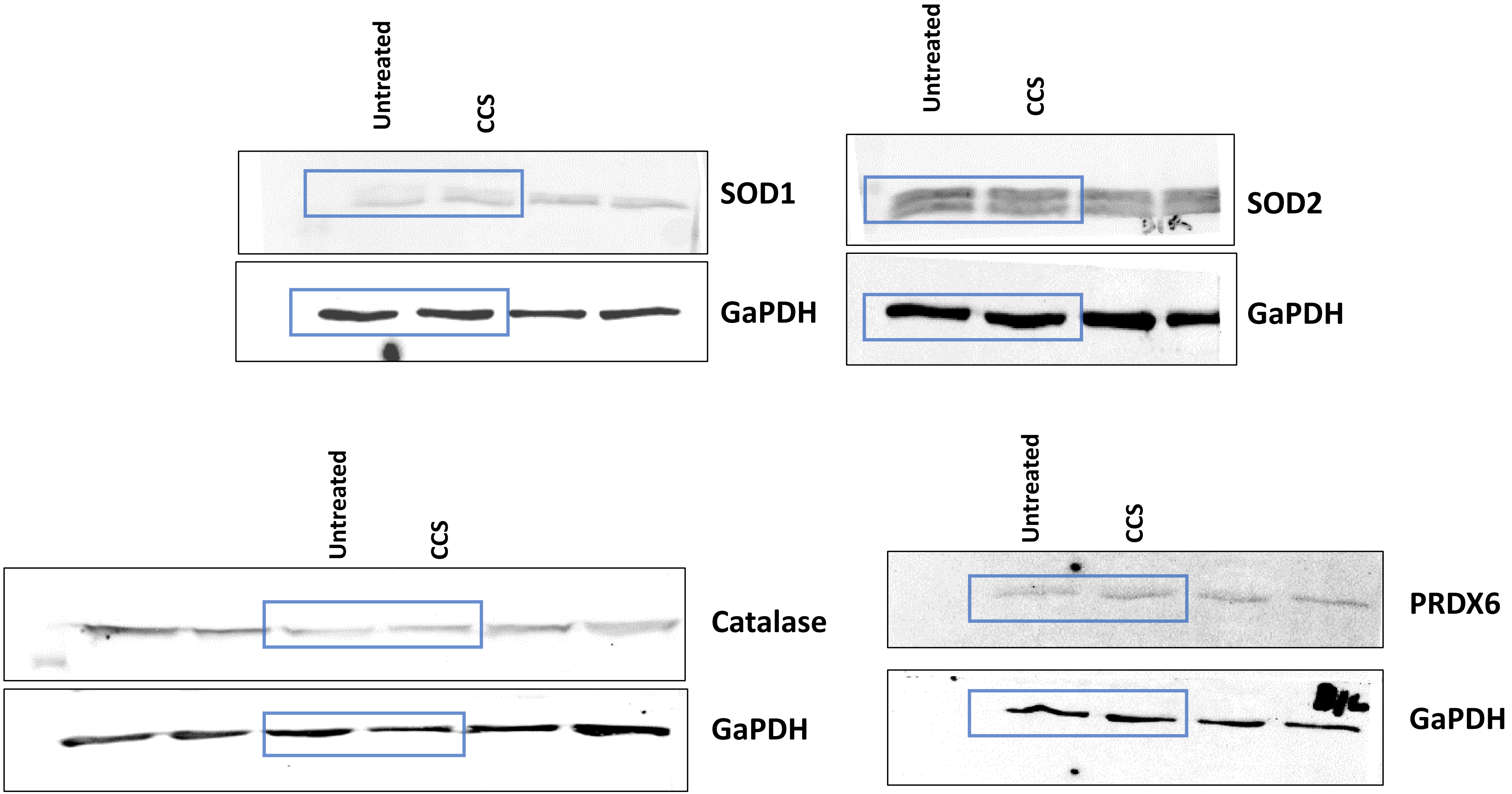

Figure S3: Original Western data of Caski-derived EVs marker proteins

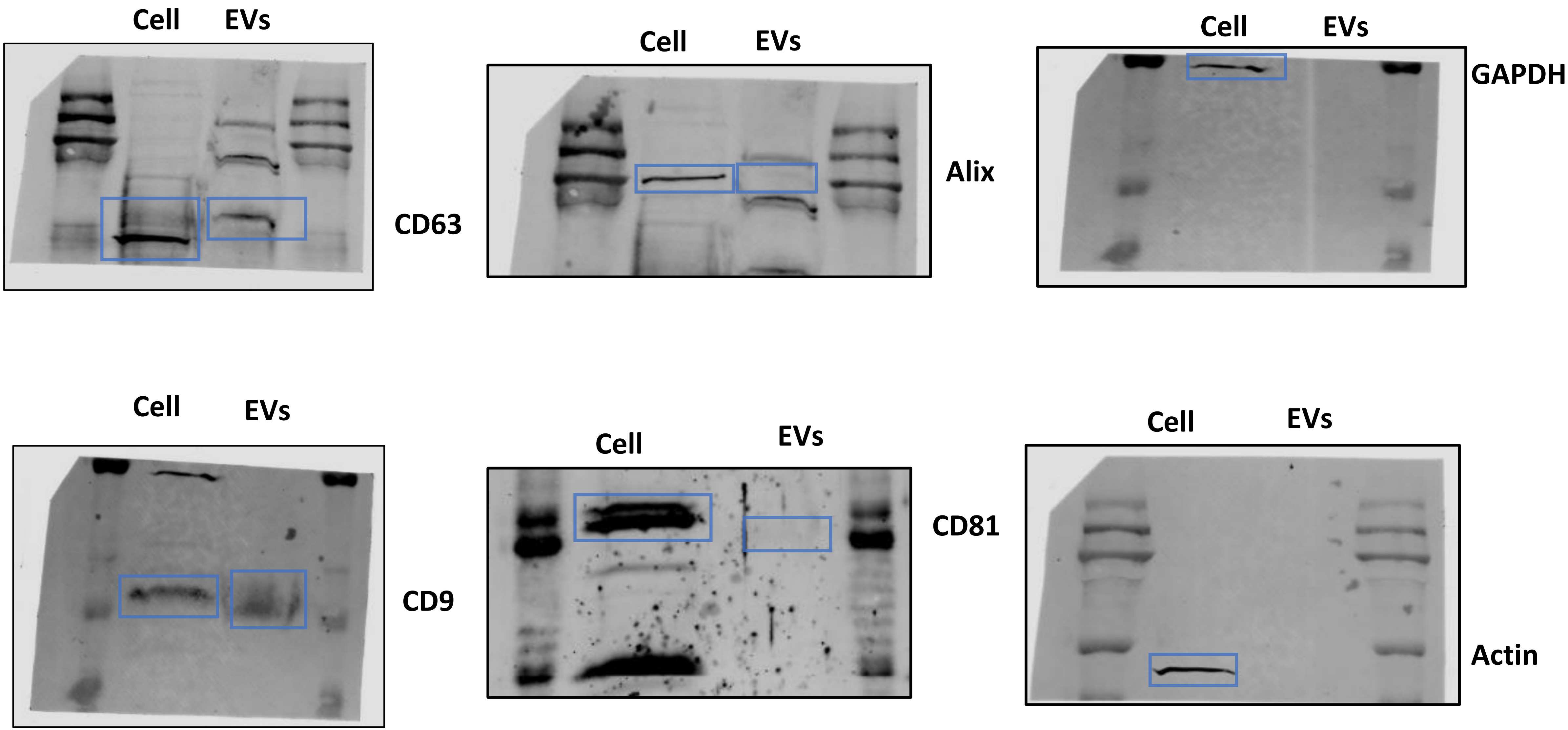

Figure S4: Original Western data of Caski cell culture-induced Evs proteins

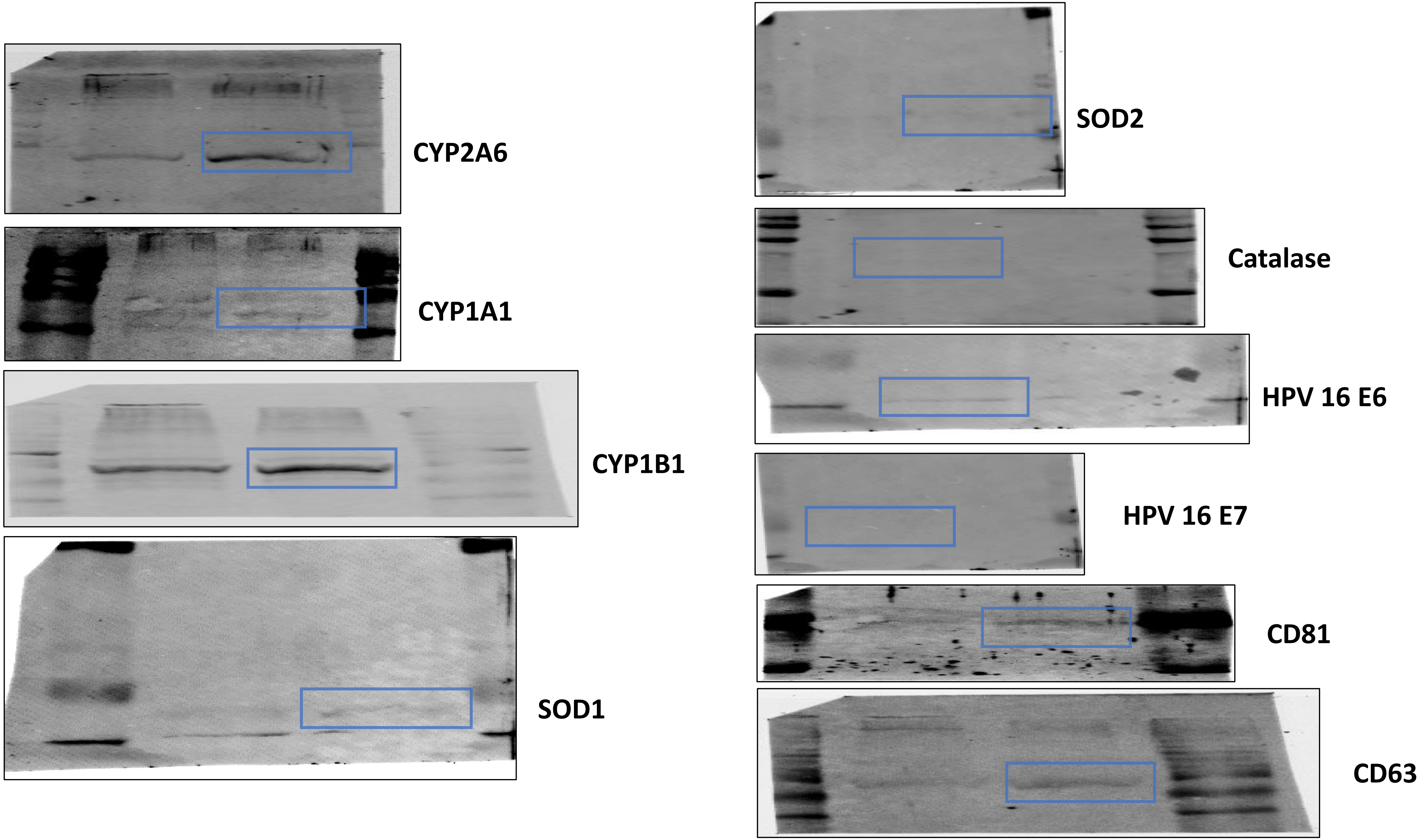

Supplement: Supplementary file 1 [file viruses-12-00239-s001.pdf]
